# From safety commitment to safety behavior: The mediating role of safety awareness

**DOI:** 10.1371/journal.pone.0332080

**Published:** 2025-12-01

**Authors:** Shanshan Lu, Weiwei Zhu, Li Zhang, Haris Ahmad

**Affiliations:** 1 School of Marxism, Hefei University of Technology, Hefei, Anhui, P.R.China; 2 School of Management, University of Science and Technology of China, Southwest University of Science and Technology, Hefei, Anhui Province, P.R. China; 3 Department of Political Science, Government Degree College Lal Qilla Maidan, Temergarah, Pakistan; University of Central Punjab, PAKISTAN

## Abstract

To deeply analyze the mechanism of workplace safety climate on employee safety behavior, a conceptual model of employee safety behavior was constructed based on the theory of stimulus-organism-response (SOR). Structural equation modeling was used to test the model and hypotheses. The results show that safety communication and safety regulation have a significant positive impact on employees’ safety commitment and, consequently, on their safety compliance behavior. However, safety communication has no significant impact on employees’ safety awareness. Safety regulation can significantly improve employees’ safety awareness, which in turn enhances their compliance behavior. Employees’ safety compliance behavior directly affects their participation behavior and plays a completely mediating role in the influence of safety commitment and safety awareness on safety participation behavior.

## 1. Introduction

The level of workplace safety has significantly improved over the last decade [1]. Employee safety behavior is crucial in reducing the occurrence of injuries and accidents. Scholars and enterprise managers have extensively studied employee safety behavior and strategies to avoid unsafe behaviors [2,3]. Employee safety behavior is closely related to the safety and health of colleagues and the public [4] and directly impacts occupational injuries and accidents [5]. Safety behavior is essential for organizational development. On one hand, it prevents physical harm; on the other hand, it positively affects the economy and society [4,6]. Current research focuses on the impacts of safety communication, safety awareness, and safety commitment on different levels of employee safety (paticipation & compliance) behavior in China. In the implementation of the “Zero Accident” initiative, safety commitment, communication, culture, and learning are key aspects [7,8]. Safety behavior is categorized into two types based on the degree of employee involvement: safety compliance behavior and safety participation behavior [9,5,10]. Safety participation behavior should be continuous rather than sporadic, as only consistent safety behavior can truly improve safety performance.

However, few studies have focused on the safety participation behavior of employees. To fill this research gap, this study examines the influence mechanism of safety climate and safety commitment on employee safety participation behavior in Chinese chemical enterprises. Drawing on the stimulus-organism-response (SOR) theory, this study clarifies how safety climate aspects (safety communication and safety regulation) impact employee’ safety perceptions (safety commitment and safety awareness) and further influence safety participation behavior. In addition to contributing to the safety management of the chemical industry, this study also contributes to the literature by discussing different levels of employee safety commitment (verbal commitment, written commitment, and behavioral commitment), which are relatively new to the safety literature. Safety behaviors can be maintained and bolstered in fire departments and companies where a positive safety climate exists [11]. From a practical perspective, this study aims to understand how to effectively maintain or increase employees’ safety participation behavior by focusing on safety commitment and safety awareness. In the long run, we discuss how to establish a good safety culture atmosphere to increase and maintain employeess’ participation behavior.

This paper is organized as follows: Section 2 presents the theoretical basis and research hypotheses, focusing on Heinrich’s causal chain theory and the Stimulus-organism-response theory. Based on safety climate and safety commitment, a series of research hypotheses are proposed. Section 3 presents the chemical enterprise employee questionnaire survey data from Shandong and Anhui provinces, China, in 2023, as well as the method and processing of data analysis. Section 4 explains the results of the statistical analyses and detailed discussion of hypotheses. Finally, the conclusion of the study is summarized, and future prospects are discussed.

## 2. Theoretical background

### 2.1. Heinrich’s causal chain theory

Heinrich’s causal chain theory [12] elucidates the relationship between various factors leading to casualty accidents and injuries. The theory introduces the concepts of unsafe behavior of people and unsafe states of objects. It emphasizes that the core of ensuring workplace safety is to prevent unsafe behavior and eliminate unsafe states. By removing any domino in the causal chain, the chain process of accidents can be interrupted, thereby preventing injury accidents [13]. The concept of the “event chain” of accident causes is constructed, and the importance of intervention in the course of accidents is clearly verified, providing a valuable method for subsequent studies of accident mechanisms [14].

### 2.2. Stimulus-organism-response (SOR) theory

Cho & Proctor [15] proposed the stimulus-response theory, which divides human behavior into two parts: stimulus and response. Further, stimulation is divided into internal stimulation from the individual and external stimulation from the environment. The SOR theory describes the entire process of behavior [16]. The stimulus is the factor that motivates people to perform behavior in the surrounding environment, the organism is the internal process transforming from stimulus to final behavior, and the response is the resulting behavior that the stimulus elicits. This theory has become one of the important frameworks for investigating the mechanism of external stimuli on individual psychology, behavior, and action [17]. Originally used to analyze the effects of the environment on human behavior, the SOR theory has been applied to many other fields, such as business and management.

## 3. Hypotheses

**3.1. Safety Communication.** The role of effective communication in safety culture has been extensively studied by scholars. [18] argued that effective communication is a hallmark of a positive safety culture within an organization. Effective communication is based on mutual trust [19]. Communication is considered a key element of a positive safety culture in health, safety, and environment (HSE) [20]. [21] argues that a typical sign of an organization’s positive safety culture is having a safety information communication system. With this system, safety information is transmitted through both formal and informal channels. [22] believe that an effective communication mechanism is important in encouraging employees to participate in safety matters, obtaining cooperation and support from employees, and maintaining a positive safety culture [23]. Based on the above, the following research hypotheses are proposed:

H1a. Safety communication is positively related to employees’ safety commitment.

H1b. Safety communication is positively related to employees’ safety awareness.

**Safety Regulation.** Safety regulation is the guideline for the safe production and business activities of enterprises [24]. However, although many enterprises have formulated a safety management system, the effectiveness of its implementation has often been unsatisfactory [25]. The safety system runs through the entire production process in coal mining enterprises, becoming a preventive measure and regulator of safety production [26]. [27] found that environmental pressure can affect the mechanism of safety accident processing. The safety system is classified as a flexible system, influenced by external factors on the cultural construction within the system.

The existing safety system still has many shortcomings, such as the lack of implementation supervision and content coherence. The overall effectiveness and credibility of the safety culture system are low, making it difficult to play the role of the system in regulating, guiding, and safeguarding [28]. The safety system, including safety regulations, still needs further improvement. Based on the above, the following hypotheses are proposed:

H2a. Safety regulation is positively related to employees’ safety commitment.

H2b. Safety regulation is positively related to employees’ safety awareness.

**Safety Commitment.** Safety commitment is not only the basic understanding of safety but also the degree of attention to safety and physical practice [29]. Safety commitment refers to employees’ ideological, emotional, and psychological identification and engagement in safety production behaviors. Employees are willing to undertake the safety responsibilities and obligations involved as members of the enterprise [30]. Relevant studies have confirmed that safety commitment is significantly related to safety atmosphere and million-ton mortality [31,10]. The key to shaping a safety culture lies in the safety commitment of managers, which determines the safety views and behaviors of general employees [32]. The degree to which an enterprise attaches importance to safety can be reflected by the visible safety commitment of managers to employees. Managers should strictly abide by the various safety management systems of the enterprise and play an exemplary role in safety behavior. Employees can judge the importance of safety management through the behavior of managers and then change their own behavior. Through interviews and on-site observations, three types of safety commitment behaviors in chemical enterprises were identified: verbal commitment of safety oath, written commitment of filling out a safety commitment letter, and behavioral commitment of wearing an exclusive safety card. Therefore, this paper focuses on the different levels of safety commitment and proposes the following hypotheses:

H3. Verbal safety commitment is positively related to employees’ safety awareness.

H4a. Written safety commitment is positively related to employees’safety compliance behavior.

H4b. Behavioral safety commitment is positively related to employees’safety participation behavior.

**Safety Awareness.** Accidents are caused by unsafe behavior of people and the unsafe state of objects, with the unsafe state of objects often being caused by human actions [33]. The root cause of people’s unsafe behavior lies in inadequate safety awareness [34]. Safety awareness is the thought that production must be safe, established in people’s minds. People are alert to external environmental conditions that may cause harm to themselves or others in production activities [35]. Safety behavior is moderated by safety knowledge and motivation [36]. Thus, the following hypotheses are proposed:

H5a. Safety awareness is positively related to employees’ safety compliance behavior.

H5b. Safety awareness is positively related to employees’safety participation behavior.

**Fig 1:**
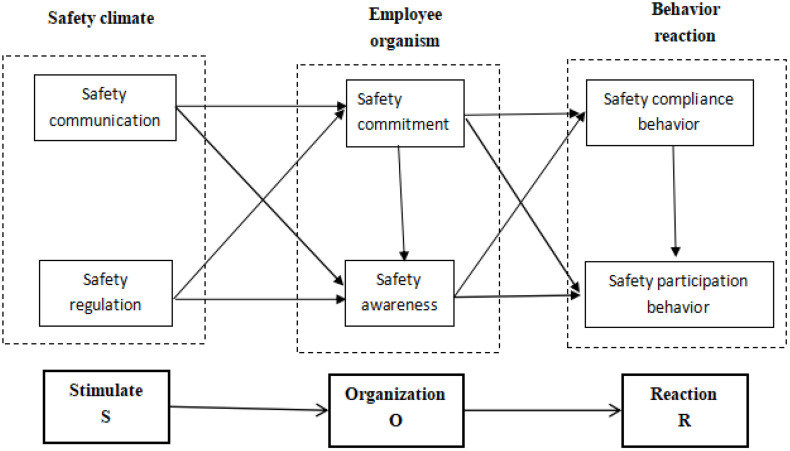
A closed-loop “S-curve” employee safety behavior model and hypotheses based on SOR theory. Safety climate & regulation (top) stimulate employee awareness and commitment (right), producing safety participation behavior (bottom), whose feedback via improved communication (left) continuously reinforces the organization’s safety system.

**Safety Behavior.** Safety behavior refers to all actions taken by individuals who comply with operating procedures during work and are able to protect themselves, equipment, and other materials in the event of a safety accident. Neal et al. [37] further clarified safety behavior and divided it into participation behavior and obedience safety behavior. Specifically, participation behavior is the behavior of employees who take the initiative to participate in the construction of workplace safety, such as helping and supervising their colleagues and giving safety advice to leaders. Obedience safety behavior is the behavior of employees who strictly abide by the rules and regulations and work according to the provisions. Safety behaviors can also be divided into safety compliance behavior and participation behavior [38]. Safety compliance behavior refers to maintaining workplace safety by carrying out basic safety activities prescribed by leaders. Safety participation behavior refers to facilitating the development of a safety-supporting environment [39]. Safety behaviors have differences at various levels and intensities. High-level safety behavior is developed gradually from low-level safety behavior. We follow the distinction between safety compliance and participation behavior, dividing employee safety behavior into participation and compliance behavior. Safety compliance behavior may impact participation behavior. The conceptual model of hypotheses is depicted in Fig 1.

H6. Employees’ safety participation behavior is positively related to the compliance behavior.

Safety climate & regulation (top) stimulate employee awareness and commitment (right), producing safety paticipation behavior (bottom), whose feedback via improved communication (left) continuously reinforces the organization’s safety system.

## 4. Method

### 4.1. Sample and data collection

To gather data and verify the proposed hypotheses, we conducted both offline and online surveys simultaneously in two chemical enterprises located in Hefei, Anhui Province, and Zibo, Shandong Province between August 1^st^ and August 30^th^, 2024. Verbal consent was obtained from participants about the distributed questionaires. Before distributing the survey, researchers conducted face-to-face interviews with the executive teams and safety officers of these enterprises. Based on feedback from professional experts, we designed and refined the final questionnaire, which included a brief introduction, measured items of constructs, and demographic descriptions.

To ensure the accuracy of the investigation results, we followed a strict process. Initially, researchers explained the significance and purpose of the investigation to the respondents. Respondents were then asked to complete the questionnaire. The online survey was conducted on the Wenjuanxing platform through Sojump.com, which distributes questionnaires via multiple terminals and channels. Before filling out the questionnaire, respondents received an informed consent form and a brief introduction to the content of the questionnaire to determine their willingness to participate. Upon confirming their participation, respondents were sent the URL link to the online questionnaire. After the survey, team members carefully reviewed the completed questionnaires. Out of 780 questionnaires collected, 175 were deemed invalid due to illogical results or missing values, leaving us with 605 valid responses. Male respondents accounted for 88.9% of the sample, reflecting the higher proportion of male employees in the chemical industry. Most respondents were middle-aged and young working-age workers: 7.3% were aged 18–25, 12.4% were aged 26–30, 38.6% were aged 31–40, and 36.4% were aged 41–50. Respondents with a junior high school degree accounted for 42.2%. The income of most respondents ranged between $9230 and $15384 yearly. The demographic characteristics of the respondents are summarized in Table 1.

**Table 1:** Demographic profile of respondents.

	Amount	Percentage (%)
**Gender**		
1. Male	538	88.9
2. Female	67	11.1
**Age**		
1. Between the ages of 18 and 25	44	7.3
2. Between the ages of 26 and 30	75	12.4
3. Between the ages of 31 and 40	234	38.6
4. Between the ages of 41 and 50	220	36.4
5. Between the ages of 51 and 60	31	5.1
6. 61 years and over	1	0.2
**Highest level of education**		
1. Lower secondary school and below	44	7.3
2. High school	239	39.5
3. Junior college	255	42.2
4. Undergraduate	64	10.5
5. Master’s degree and above	3	0.5
**Annual personal income (RMB)**		
1. Less than 30,000	26	4.3
2. 30,000–60,000 (excluding 60,000)	123	20.4
3. 60,000–100,000 (excluding 100,000)	413	68.2
4. 100,000–150,000 (excluding 15,000)	30	4.9
5. More than 150,000	13	2.2
**Residence**		
1. Urban district	341	56.3
2. Villages and other areas	264	43.7

### 4.2. Measures

All constructs in the research model were evaluated using three to five items derived from prior studies, with some items modified to fit the context of this study. We used a five-point Likert scale ranging from “strongly agree” to “strongly disagree.” The measurement items for safety climate (safety communication and safety regulation) were adopted from [31]. Safety commitment was assessed using six items from [40]. Risk awareness was measured using a multi-item scale, including responsibility awareness and rule awareness. The items for safety compliance and participation behavior were adopted from [39]. Adjustments and improvements were made based on the actual work scenarios of employees in the chemical industry.

**Safety communication.** Five items were used to measure employee safety communication at workplace. Employees answered the following questions: “As a group, we constantly remind each other how to work safely”, “My colleagues and I were able to communicate safety information to each other effectively”, “I can communicate safety information effectively with my colleagues in other teams or departments”, “My supervisor and I were able to communicate safety information to each other effectively”, “We have regular safety talks in workplace”.

**Safety regulation.** To measure safety regulation in the enterprise, six relevant questions were asked: “The current safety regulation of the enterprise is rigorous and well-formulated”, “The current safety regulation of the enterprise can ensure the orderly implementation of safe production”, “The current safety regulation of enterprises has certain deficiencies and defects”, “The current safety regulation helps me do my job successfully”, “I am very familiar with the current Safety regulation in the enterprise”, “I am very satisfied with the current Safety system in the company”.

**Safety commitment.** As for the measurement of employee safety commitment, six items were used. Specifically: “I will make verbal commitments at work (e.g., safety oath)”, “I will make a written commitment at work (such as: safety responsibility letter)”, “I will make behavioral commitments at work (e.g., wear a Safety commitment card)”, “I think a commitment to safety at work is important”, “I think a commitment to safety is very essential at work”, “I appreciate the commitment to safety at work”.

**Safety awareness.** Four items were designed to measure employee safety awareness. Specific issues include: “I think all accidents are preventable”, “I have a strong sense of safety at work”, “I think safety awareness is very important at work”, “When possible safety risks are found, I will deal with them and report them in time”.

**Safety compliance behavior.** Employees’ safety compliance behavior is a passive, temporary safety behavior that lacks continuity and long-term effectiveness. The measurement of compliance behavior is measured using following question items. “I will use the essential safety equipment to do my job”, “I will follow the correct safety procedures to do my job”, “I will do my job in a safe way and with safe habits”, “I will ensure the highest level of safety in the completion of my work”.

**Safety participation behavior.** Differently, employees’ safety behavior is a proactive, long-term safety behavior that is continuous and has lasting effects. To measure participation behavior, four specific items were asked. “I volunteer to perform tasks or activities that contribute to improving workplace safety”, “I will make an extra effort to improve workplace safety”, “I will actively share and publicize safety knowledge”, “I will give advice on the safety culture and system of the company”. Table 2 presented the constructs and measurement items.

**Table 2:** Constructs and measurement items. [39].

Constructs	Number of items	Measurement items	Source
Safety communication (SC)	5	As a group, we constantly remind each other how to work safely. My colleagues and I were able to communicate safety information to each other effectively.	[31]
		I can communicate safety information effectively with my colleagues in other teams or departments.	
		My supervisor and I were able to communicate safety information to each other effectively.	
		We have regular safety talks in workplace.	
Safety regulation (SR)	6	I have a good understanding of the organization’s current safety regulation. The current safety regulation of the enterprise is scientifically advanced.	
		The enterprise’s current safety regulation ensures that safe production is organized.	
		Certain deficiencies and shortcomings in the current safety regulation of the enterprise.	
		The current safety regulation of the enterprise contributes to the successful completion of my work.	
		I am very satisfied with the current Safety system in the company.	
Safety commitment (ST)	6	I will make verbal commitments at work (e.g., safety oath).	
		I will make a written commitment in my work (e.g., a statement of security responsibilities).	
		I will make behavioral commitments at work (e.g., wear a safety commitment card).	
		I believe that a commitment to safety at work is important for safe production. I think a commitment to safety is very essential at work. I appreciate the commitment to safety at work.	
Safety awareness (SA)	4	I believe that all accidents are preventable. I have a strong sense of safety at work.	
		I believe that safety awareness is very important in my work.	
		I’ll take my chances when it comes to identifying possible safety hazards.	
Safety complicance behavior (SCB)	4	I will use the essential safety equipment to accomplish my work.	
		I will follow proper security procedures to complete my work.	
		I will perform my work in a safe manner and behavioral habits.	
		I will perform my job with the highest level of safety.	
Safety paticipation behavior (SPB)	4	I will make an extra effort to improve workplace safety. I will actively share my safety knowledge and publicize it (e.g., by making safety micro-videos).	[39]
		I will contribute to the organization’s safety culture and system. I will give advice on the safety culture and system of the company.	

**Control variables.** Several potential factors that may impact employees’ safety behaviors were considered as control variables. We controlled for employee demographics, such as age, gender, years of work experience, and education. We also controlled for employees’ accident experience. Lastly, we excluded the impact of risk perception and safety information attentiveness.

### 4.3. Data analysis and results

Structural equation modeling (SEM) was utilized to conduct measurement model testing and to verify the research hypotheses. The constructs described in the research model were measured using multiple items, and structural relationships exist between these constructs.

**Basic model test.** Confirmatory factor analysis (CFA) was performed using AMOS 21.0 software to test the model and evaluate the independent structure. The full model includes workplace safety climate (safety communication, safety regulation), employee reactions and perceptions (safety commitment, safety awareness), and employee safety behaviors (compliance behavior, participation behavior). The goodness-of-fit test results are shown in Table 3. The model fits well according to the results and is in line with expectations.

**Table 3:** Model fitting index results.

Index	x^2^/df	SRMR	RMSEA	NFI	TLI	GFI	CFI
**Evaluation Standard**	1.523	0.002	0.030	0.988	0.914	0.963	0.989
**Index value**	<5	<0.08	<0.1	>0.8	>0.8	>0.8	>0.8

**Measurement model test.** In the measurement model testing, the reliability and validity of the constructs and measurement items were evaluated using CFA. Table 2 shows the results of the CFA. Cronbach’s alpha values for the variables were above 0.970, and the composite reliability values ranged from 0.89 to 0.95. These scores are all higher than the suggested threshold value of 0.70 [41]. The results indicate that the internal consistency of the measurement items for each construct is good, and the reliability of the measurement model is adequate.

Convergent validity and discriminant validity were both assessed to ensure that the measurement items successfully evaluated the corresponding constructs. The loadings of the measurement items and the average variance extracted (AVE) were analyzed to test convergent validity. Table 1 shows that the minimum value of AVE is 0.899, which meets the requirements. Moreover, the loadings for each item are above the reference value of 0.70. Overall, the evaluation model has adequate convergent validity. Three guidelines were used to verify discriminant validity: the loading of each item for all constructs should be higher than cross-factor loadings. The correlation values between each pair of constructs should not exceed 0.85 [42]. The square root of the AVEs should be larger than inter-construct correlations. As shown in Table 4, the loadings of all elements for the corresponding measured construct are higher than the cross-factor loadings of items for other constructs.

**Table 4:** Reliability test of the scale.

Scales	Name of variables	Number of question items	Cronbach’s α	AVE
**Safety climate** **(Stimulus)**	Safety communication (SC) Safety regulation (SR)	5 6	0.990 0.989	0.952 0.899
**Employee consciousness** **(Organism)**	Safety commitment (ST) Safety awareness (SA)	6 4	0.988 0.970	0.945 0.919
**Employees’ behavior (Response)**	Safety compliance behavior (SCB) Safety participation behavior (SPB)	4 4	0.987 0.984	0.961 0.953

**Fig 2:**
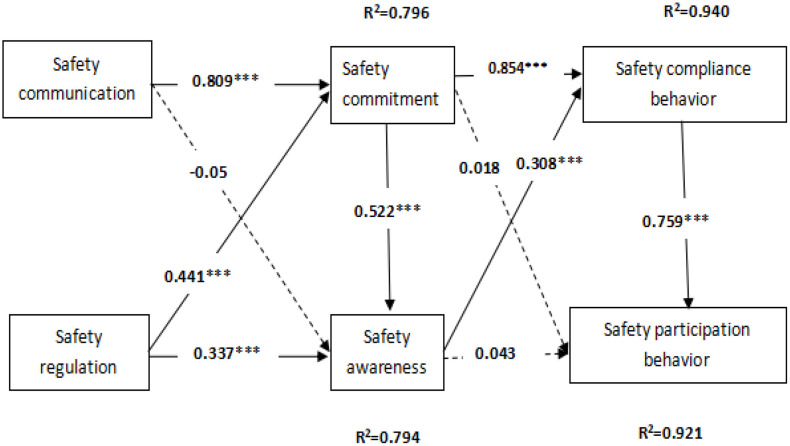
Result of the structural model testing. Safety regulation drives safety participation behavior (0.759) and compliance behavior (0.308); both enhance safety commitment and safety commitment (0.522, 0.441), which strongly predicts safety behavior (0.854, R^2^ = 0.940), while safety communication shows no significant effect.

**Structural model test.** Hypotheses testing on the basic model and the standardized path coefficients and significance tests were conducted. The results of the coefficients are as follows: Safety communication can predict safety commitment (β = 0.809, p < 0.001), but it has no significant impact on safety awareness (β = −0.05, p > 0.05). Safety regulation has a positive effect on both safety commitment (β = 0.441, p < 0.001) and safety awareness (β = 0.337, p < 0.001). Safety commitment can predict safety awareness (β = 0.522, p < 0.001) and safety compliance behavior (β = 0.854, p < 0.001), but it does not significantly predict safety participation behavior (β = 0.018, p > 0.05). Similarly, safety awareness has a positive impact on safety compliance behavior (β = 0.308, p < 0.001), but it does not significantly impact safety participation behavior (β = 0.043, p > 0.05). Safety compliance behavior can predict participation behavior (β = 0.759, p < 0.001). The results are shown in Fig 2.

Safety regulation drives safety participation behavior (0.759) and compliance behavior (0.308); both enhance safety commitment and safety commitment (0.522, 0.441), which strongly predicts safety behavior (0.854, R^2^ = 0.940), while safety communication shows no significant effect.

The proposed research hypotheses were verified by examining the results of the data analysis. The overall model demonstrates that safety communication has a significant positive impact on safety commitment, supporting Hypothesis 1a (H1a). However, safety communication does not significantly affect safety awareness, leading to the rejection of Hypothesis 1b (H1b). Safety regulation positively influences both safety commitment and safety awareness, thereby supporting Hypotheses 2a and 2b (H2a-H2b). Safety commitment has a positive effect on safety awareness, confirming Hypothesis 3 (H3). Safety commitment significantly predicts safety compliance behavior but not participation behavior, supporting Hypothesis 4a (H4a) while rejecting Hypothesis 4b (H4b). Similarly, safety awareness positively affects safety compliance behavior but not participation behavior, supporting Hypothesis 5a (H5a) and rejecting Hypothesis 5b (H5b). Lastly, safety compliance behavior is found to predict participation behavior, confirming Hypothesis 6 (H6). The detailed results of the hypothesis tests are presented in Table 5.

**Table 5:** Significance test results of the basic model.

Hypotheses	Path	Standardized path coefficients	t value	Test result
**H1a**	Safety communicationSafety commitment	0.809***	8.332	Pass
**H1b**	Safety communicationSafety awareness	−0.05	−0.582	Fail
**H2a**	Safety regulation Safety commitment	0.441***	6.197	Pass
**H2b**	Safety regulation Safety awareness	0.337***	4.077	Pass
**H3**	Safety commitment Safety awareness	0.522***	6.787	Pass
**H4a**	Safety commitment Safety compliance behavior	0.854***	9.306	Pass
**H4b**	Safety commitment Safety participation behavior	0.018	0.230	Fail
**H5a**	Safety awareness Safety compliance behavior	0.308***	3.301	Pass
**H5b**	Safety awareness Safety participation behavior	0.043	0.436	Fail
**H6**	Safety compliance behavior Participation behavior	0.759***	7.291	Pass

Note: *** means p < 0.001, ** means p < 0.01, * means p < 0.05.

### 4.4. Mediating effect test

The results of the hypotheses testing indicate that safety commitment not only directly influences safety behaviors but also indirectly impacts them through safety awareness. In contrast, safety commitment and safety awareness have a direct effect on safety compliance behavior but not on participation behavior. Therefore, this study explores the mediating effect of safety awareness through the following three steps: First, the influence of the independent variable (safety commitment) on the dependent variable (safety behavior) is examined and should be significant. Second, the mediator (safety awareness) should be significantly impacted by the independent variable (safety commitment). Finally, both the independent variable and the mediator are included in a regression model to assess their effects on the dependent variable. If the dependent variable is significantly determined by the mediator but not by the independent variable, a complete mediating effect of the mediator is suggested. Conversely, if both the mediator and the independent variable significantly determine the dependent variable, a partial mediating effect is indicated. If the effects in either the first or second step are insignificant, the mediator is deemed to have no mediating effect.

Table 6 presents the results of the mediating effect test based on the above principles. For safety compliance behavior, the direct influence of safety commitment is significant (β = 0.448, p < 0.001), and the indirect effect of safety commitment mediated by safety awareness is also significant (β = 0.339, p < 0.001). These results suggest that safety awareness partially mediates the impact of safety commitment on compliance behavior, accounting for 26.5% of the effect.

**Table 6:** Mediating effect test for employee safety (Compliance/ Participation) behavior.

Path	Effect	Point Estimate	Product of Coefficient		
			S.E.	Est./S.E.	p-Value
ST-SA- SCB	Indirect Direct	0.448*** 0.339***	0.035 0.026	4.235 2.576	0.000 0.000
SR-ST- SCB	Indirect Direct	0.731*** 0.137***	0.056 0.022	7.942 0.727	0.000 0.000
SR-SA- SCB	Indirect Direct	0.384*** 0.484***	0.050 0.029	3.178 4.425	0.000 0.000
SC-ST-SPB	Indirect Direct	0.631*** 0.173***	0.051 0.023	5.826 1.043	0.000 0.000
ST-SA- SPB	Indirect Direct	0.423*** 0.382***	0.035 0.026	4.009 3.040	0.000 0.000
SR-ST- SPB	Indirect Direct	0.585*** 0.299***	0.069 0.027	5.481 1.989	0.000 0.000
SR-SA- SPB	Indirect Direct	0.342*** 0.543***	0.049 0.029	2.733 5.074	0.000 0.000

Note: *** means p < 0.001, ** means p < 0.01, * means p < 0.05.

However, the direct influence of safety commitment on participation behavior is not significant (β = 0.018, p > 0.05), indicating that the mediating effect of safety awareness on participation behavior is not supported. This may be because safety awareness alone may not be sufficient to stimulate an increase in participation behavior. Future research should consider the combined effects of safety commitment and safety awareness.

## 5. Discussion

### 5.1. Theoretical implications

Several theoretical contributions can be summarized from this research. This study systematically investigates the influencing factors and formation mechanisms of safety participation behavior among employees in the chemical industry. First, the Stimulus-Organization-Reaction (SOR) model is expanded. Awareness is highlighted as a crucial element in explaining behavioral reactions. Similarly, this study focuses on safety awareness and extends the SOR model by incorporating additional variables, namely safety communication, safety regulation, and safety commitment. These three factors are interrelated and have a significant impact on safety behavior. Moreover, the formation mechanism of employee safety participation behavior is explained in detail.

### 5.2. Practical implications

The results of this study provide practical implications for improving safety participation behavior among employees in the chemical industry. The findings can help encourage employee safety behavior and reduce production accidents in China. Specifically, the study offers several recommendations for business managers and policymakers:Unsafe behavior is proven to have a direct effect on safety participation behavior. Therefore, efforts should be made to improve employees’ unsafe behavior in the initial stage. Operating procedures and norms should be clarified in the workplace. In addition, the safety awareness of employees should be improved. Two examples of how enterprises can embed the findings into daily safety protocols are as follows: On the one hand, add the Pre-Task Commitment Cards. Require every work team to fill out a pocket-sized card that states: (a) the critical step, (b) the personal protective action they commit to, and (c) the colleague who will verify it. Cards are photographed and uploaded to the HSE dashboard;completion rates are tracked in real time. Furthermore, actively organizing Regulation-to- Action Huddles is also a good choice. Within 24 h of any new safety regulation, supervisors conduct a 5-minute floor huddle using a laminated “Regulation-to-Action” template that translates the rule into three observable behaviors. Compliance is checked during the next routine audit. Authorities should strive to enhance workers’ safety commitment through measures such as safety oaths, safety responsibility letters, and safety commitment cards. Promoting Safety Communication is also important. Safety communication has a positive influence on safety commitment, which means reducing communication barriers is essential. Specifically, creating a culture and comprehensive communication channels that support barrier-free communication within the enterprise is crucial. Safety regulation is crucial to both safety commitment and awareness. Business managers can establish top-down regulations for employee safety behavior, including reward and punishment systems. Government departments should enact effective laws and regulations on occupational safety, and industry experts should provide guidance and training to reduce accidents. Government officials should continue to strengthen publicity efforts regarding severe production accidents and increase attention to safety awareness and responsibility through multiple channels. For enterprises, it is meaningful to establish a secure platform to help employees understand the risks of unsafe behavior. Strengthening safety legislation remains necessary to further improve employees’ safety awareness and behaviors.

### 5.3. Limitations and prospects

This study enriches the research on employee safety behavior from both theoretical and practical aspects. Despite obtaining meaningful results for safety culture and behaviors, the present study contains several limitations, which provide opportunities for future research. First, given the difficulty of obtaining data on real behavior, this study measured potential behavioral reactions rather than actual actions. Future research could explore the key predictors and influencing mechanisms of employee safety participation behavior through a two-stage investigation. Specifically, common-method bias may inflate the observed relationships because all measures were collected via the same self-report questionnaire at a single point in time. To overcome these limitations, we plan to collect multi-wave data (e.g., baseline, 3-month, and 6-month follow-ups) to examine the temporal ordering of the variables and to test reciprocal effects. Second, the expanded SOR model is presented as the conceptual foundation to verify the mechanism of employee behaviors. Future research could focus on the safety behavior mechanisms of leaders and the influencing factors.Third, the present study focuses on the chemical industry in China. Future studies could explore differences across different regions and industries.

## 6. Conclusions

This research provides an in-depth investigation into how safety stimulus factors influence employee safety behavior, including safety communication and regulation, safety commitment, and safety awareness. The study explores the crucial influencing factors and explains the underlying mechanisms based on the extended model of stimulus-organization-reaction.

The findings indicate that safety communication can enhance employees’ safety commitment, while safety regulation has a positive impact on both safety commitment and safety awareness. Safety commitment also significantly influences safety awareness. Both safety awareness and safety commitment play significant roles in promoting unsafe behavior. Furthermore, safety awareness partially mediates the impact of safety commitment on unsafe behavior. However, safety commitment and safety awareness do not directly trigger safety participation behavior. Instead, they indirectly influence participation behavior by affecting unsafe behavior. In other words, safety awareness and commitment can only improve unsafe behavior rather than directly enhancing participation behavior. Unsafe behavior serves as a starting point and foundation for participation behavior. The improvement ofparticipation behavior is not achieved overnight; it requires persistence and continuous effort.
